# PBDEs Found in House Dust Impact Human Lung Epithelial Cell Homeostasis

**DOI:** 10.3390/toxics10020097

**Published:** 2022-02-19

**Authors:** Antonio Zandona, Karla Jagić, Marija Dvoršćak, Josip Madunić, Darija Klinčić, Maja Katalinić

**Affiliations:** Biochemistry and Organic Analytical Chemistry Unit, Institute for Medical Research and Occupational Health, HR-10001 Zagreb, Croatia; azandona@imi.hr (A.Z.); kjagic@imi.hr (K.J.); msambolec@imi.hr (M.D.); jmadunic@imi.hr (J.M.)

**Keywords:** PBDEs, A549, house dust, cytotoxicity, viability, prolonged exposure

## Abstract

The toxicity of eight polybrominated diphenyl ethers (PBDEs) congeners detected in environmental and biological samples (BDE-28, -47, -99, -100, -153, -154, -183, and -209) was evaluated on the epithelial lung cells. Exposure to these PBDEs increased membrane disruption and a release of lactate dehydrogenase, accompanied by oxidative stress in cells through the formation of reactive oxygen species (ROS) and a decrease in mitochondrial membrane potential. Interestingly, some of the tested PBDEs increased apoptotic markers as well. For several congeners, the observed toxicity was time dependent, meaning that even smaller concentrations of these compounds will have negative effects over time. Such time-dependent toxicity was also confirmed for cell treatment with a real house dust sample extract. This could be indicative with regard to the constant exposure to a mixture of PBDE congeners through different pathways in the organism and thereby presenting a risk for human health. As such, our findings point to the importance of further studies on the negative effects of PBDEs to understand their mechanism of action in detail.

## 1. Introduction

**Figure 1:**
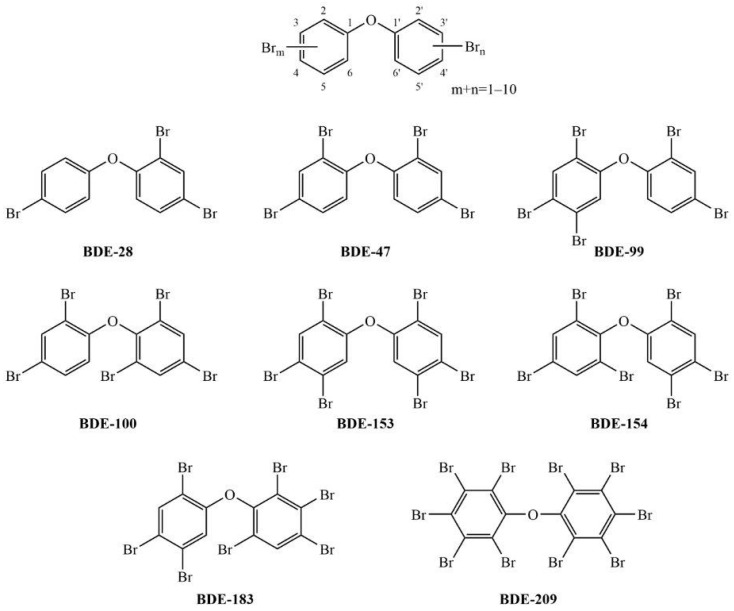
The general structure of polybrominated diphenyl ethers and the structure of the eight environmentally relevant congeners tested in this study.

Polybrominated diphenyl ethers (PBDEs, Figure 1) were extensively used as chemical additives to textiles, furniture, building materials and electrical/electronic devices to reduce their flammability and prevent or slow down potential fires [1]. As they do not form a chemical bond with the material, they are easily detached from household products and adsorbed onto the organic particles of house dust. Although their use has been completely banned for several years now, massive reserves of products containing PBDEs and enhanced recycling alongside their persistence result in continuous human exposure. Ingestion of contaminated food, dust and air particles, dermal absorption of dust particles along with inhalation of contaminated air, air particles and dust particles are the main exposure routes for humans [2,3,4]. Two population groups are most exposed to PBDEs: infants consuming mothers’ milk and toddlers due to their hand-to-mouth behavior and extensive contact with dusty surfaces. Epidemiological studies suggest an association between PBDE exposure (both prenatal and postnatal) and a lower birth weight, lower levels of thyroid-stimulating hormone, lower intelligence quotient, increased incidence of hyperactivity disorder and an impaired cognitive, motor and behavioral neurodevelopment [5].

At the cellular level, it has been shown that several PBDEs can have various negative effects, including reduction in cell viability, induction of oxidative stress, apoptosis, mitochondrial dysfunction and DNA damage [6,7,8,9,10,11,12,13,14,15,16]. Assessments of the effects of PBDEs on the human respiratory system are scarce and limited to only three congeners: BDE-47, -99 and -209 [9,14,17,18]. However, a recently analyzed house dust sample from Zagreb, Croatia, pointed to the presence of several other congeners contributing to higher overall PBDE concentrations [19]. In the most contaminated dust sample, the sum of seven PBDE congeners exceeded 17 µg g^−1^ dust. Therefore, the aim of our study was to examine the effects of detected, less studied PBDE congeners, namely BDE-28, -100, -153, -154 and -183 on human cells (human carcinoma alveolar basal epithelial cells A549). The most researched congeners BDE-47, -99 and -209, known to trigger diverse effects [6,8,9,18], were also included in this study for comparison. The idea was to examine more closely the impact of exposure by measuring oxidative and antioxidative status, mitochondrial membrane potential, integrity of cell membrane and induction of apoptosis, since disturbances in these systems can severely damage cell function or lead to cell death. 

## 2. Materials and Methods

### 2.1. Chemicals

Commercial standards of BDE-28 (2,4,4′-tribromodiphenyl ether), BDE-47 (2,2′,4,4′-tetrabromodiphenyl ether), BDE-99 (2,2′,4,4′,5-pentabromodiphenyl ether), BDE-100 (2,2′,4,4′,6-pentabromodiphenyl ether), BDE-153 (2,2′,4,4′,5,5′-hexabromodiphenyl ether), BDE-154 (2,2′,4,4′,5,6′-hexabromodiphenyl ether), BDE-183 (2,2′,3,4,4′,5′,6-heptabromodiphenyl ether) and BDE-209 (decabromodiphenyl ether) (Figure 1) at a concentration of 50 µg mL^−1^ in *n*-nonane were purchased from LGC Standards GmbH (Wesel, Germany).

Each PBDE congener was reconstituted in methanol (J. T. Baker, Derventer, The Netherlands) at a concentration of 500 µg mL^−1^ and diluted further for experimental purposes. An extract of real house dust in *n*-hexane containing a total PBDE concentration of 17 µg mL^−1^ [19] was also reconstituted in methanol to obtain a final concentration of 340 µg mL^−1^ and was diluted further, according to the needs of the experiment.

### 2.2. Cell Culture

The certified human carcinoma alveolar basal epithelial A549 cell line (ECACC 86012804) was purchased from the European Collection of Authenticated Cell Cultures (ECACC) cell bank. Cells were grown in RPMI-1640 medium (Sigma-Aldrich, Steinheim, Germany) supplemented with 10% (*v*/*v*) fetal bovine serum (FBS, Sigma-Aldrich, Steinheim, Germany) and 1% (*v*/*v*) penicillin/streptomycin (PenStrep, Sigma-Aldrich, Steinheim, Germany). The A549 cells were grown at 37 °C in a 5% CO_2_ atmosphere; the medium changed every few days, and passages were conducted according to the standard protocol (ECACC, 2018).

Phosphate-buffered saline (PBS, pH 7.4) was prepared according to a standard procedure [20] and used for washing the cells when needed in the assays.

### 2.3. Measurement of Cell Viability (MTS)

The cytotoxic profiles of the tested PBDEs were determined by measuring the succinate dehydrogenase mitochondrial activity of the cells exposed to them [21]. We used the commercially available MTS detection reagent assay (CellTiter 96^®^ AQueous One Solution Cell Proliferation Assay, G3582, Promega, Madison, WI, USA), following a previously described protocol [22]. Following that protocol, the cells were seeded into 96-well plates at a density of 20,000 cells/well one day before the experiment. The initial number of 20,000 cells/well was seeded, according to previous experience and literature data [23,24], and it is thought to be sufficient for covering 96-well plate well bottom. Due to the doubling time of A549 cells, it is expected to reach a maximum of 80,000 cell/well within 72 h of being tested, which is in accordance with the used MTS dye manufacturer’s protocol. In other words, according to them, the measurement can be performed without signal saturation up to 160,000 cells/well.

The method was validated with positive control staurosporine (0.03–4 µM final concentration), a known inductor of apoptosis [25], according to the manufacturer’s guidance. Furthermore, cells were inspected under the microscope before the experiment and during the experiment every day to verify correct confluence.

On the day of the experiment, a wide concentration range of PBDEs (0.375–48 µg mL^−1^) was added to the complete medium supplemented with 10% of FBS, and cells were continuously exposed for 24, 48 and 72 h with no medium changes. The highest percentage of methanol did not influence cell viability. The medium was added at a sufficient volume to ensure the availability of all nutrients through 72 h. After the desired time of incubation at 37 °C in a 5% CO_2_ atmosphere, the cells were washed with PBS buffer, and 100 µL of RPMI-1640 and 20 µL of MTS were added to each well. After the addition of MTS dye, color development was monitored continuously visually until the first changes were observed, normally within 1–4 h, and then the changes were quantified spectrophotometrically. Therefore, color saturation did not occur, irrespective of the number of cells/well.

After a designated time, the absorbance was read at 492 nm on an Infinite M200PRO plate reader (Tecan Austria GmbH, Salzburg, Austria). We evaluated the means of three separate experiments (each conducted in duplicate) and presented them as percentages of either the counted viable cells or of inhibited cells (obtained by deducting the counted viable cells from 100%) compared to the untreated cells as a control, i.e., IC_50_ values were determined from cytotoxicity curves with a nonlinear fit equation from the Prism 8 software (GraphPad Software, San Diego, CA, USA).

### 2.4. Cell Membrane Integrity

Cell membrane integrity was determined by measuring the release of the intracellular LDH from cells with a damaged membrane into the medium. Namely, as described in our previous study cited here [23], cells were seeded at a density of 20,000 cells/well in 96-well black plates and were exposed to PBDEs at three concentrations (3, 6 and 12 µg mL^−1^) for 4 h. Compounds were prepared in the unsupplemented medium without FBS. After a 3.5 h incubation at 37 °C in a humidified atmosphere containing 5% CO_2_, cells were equilibrated to 22 °C for 30 min. An amount of 100 µL/well of the reagent (1:1, The CytoTox-ONE™ Homogeneous Membrane Integrity Assay, G7891, Promega, Madison, WI, USA) was added to each well, according to the manufacturer’s protocol, shaken for 30 s and incubated at room temperature protected from light for 10 min. Fluorescence was recorded on an Infinite M200PRO plate reader (Tecan Austria GmbH, Salzburg, Austria) or SynergyMx (BioTek Instruments, Winooski, VT, USA) with an excitation wavelength of 560 nm and an emission wavelength of 590 nm. Triton X-100 at a final concentration of 0.18% (*v*/*v*) (9% in water stock; Sigma-Aldrich, Steinheim, Germany) was used as a positive control to determine maximal LDH release. Data were evaluated from at least two or three independent experiments (performed in duplicate or triplicate) using Prism 6/8 software and presented as a percentage of LDH release compared to the determined maximal LDH release. Calculations were performed according to the predefined equation from CytoTox-ONE™ Homogeneous Membrane Integrity Assay kit protocol.

### 2.5. Determination of Reactive Oxygen Species

Reactive oxygen species (ROS) were determined using a cell-permeable reagent 2′,7′–dichlorofluorescin diacetate dye (DCFDA, Sigma-Aldrich, Steinheim, Germany). One day before the experiment, the A549 cells were seeded into 96-well black plates at a density of 20,000 cells/well, and on the day of the experiment, incubated with the PBDEs at the concentrations of 3, 6 and 12 µg mL^−1^ in the unsupplemented medium without FBS at 37 °C in a 5% CO_2_ atmosphere for 4 h and then removed and washed once with PBS 100 µL/well. Then, 100 µL of DCFDA was added to each well to obtain the final concentration of 50 µM [23]. The plate was incubated for 30 min in the dark, after which fluorescence was recorded on an Infinite M200PRO plate reader with excitation and emission spectra of 495 nm and 529 nm, respectively. For positive control, we used hydrogen peroxide (H_2_O_2_, Sigma-Aldrich, Steinheim, Germany) at the final concentration of 100 µM. We evaluated the mean of three separate experiments (each conducted in duplicate) using the Prism software and presented as normalized signal to untreated control, according to the dye manufacturer’s calculation protocol.

### 2.6. Determination of Glutathione

Intracellular glutathione (GSH) was determined by quantifying its conjugation with cell-permeable reagent monochlorobimane (mCB, Sigma-Aldrich, Steinheim, Germany), forming a fluorescent signal. The cells were prepared and incubated with PBDEs, as described above, for ROS measurements. Then, 100 µL of mCB dye was added to each well to get the final concentration of 40 µM. The plate was incubated for 20–40 min, and then fluorescence was recorded on an Infinite M200PRO plate reader with an excitation and emission spectra of 355 nm and 460 nm, respectively. For positive controls, we used tert-butyl hydroperoxide (tBOOH, Sigma-Aldrich, Steinheim, Germany) at the final concentration of 100 µM. We evaluated the mean of three separate experiments (each conducted in duplicate) using the Prism software and presented as normalized signal to untreated control, according to the dye manufacturer’s calculation protocol.

### 2.7. Determination of Mitochondrial Membrane Potential

Mitochondrial membrane potential (ΔΨ_m_) was determined using cell-permeable potential-sensitive cationic dye tetramethylrhodamine ethyl ester perchlorate (TMRE) from the assay kit (Mitochondrial Membrane Potential Assay Kit (II), #13296, Cell Signaling Technology Europe, Leiden, The Netherlands). Namely, as described in our previous study cited here [23], cells were prepared and incubated with PBDEs, as described above, for ROS and GSH measurements. Each well received 10 µL of 2 µM TMRE in culture medium, and the plate was incubated for another 20 min. The medium was aspirated, cells washed three times with PBS, and 100 µL of PBS was added to each well. Fluorescence was recorded on an Infinite M200PRO plate reader with excitation and emission spectra of 550 nm and 580 nm, respectively. For positive control we used carbonyl cyanide 3-chlorophenylhydrazone (CCCP, Cell Signaling Technology Europe, Leiden, The Netherlands) at the final concentration of 50 µM. We evaluated the mean of three separate experiments (each conducted in duplicate) using the Prism software and presented as normalized signal to untreated control, according to the manufacturer’s calculation protocol.

### 2.8. Apoptosis

PBDE-mediated induction of apoptosis was analyzed by detecting the translocation of phosphatidylserine (PS) to the cell surface using an Annexin V probe and following loss of membrane integrity using 7-aminoactinomycin D (7-AAD) dye. For this purpose, we used predefined Muse^®^ Annexin V & Dead Cell Assay (MCH100105, Merck KGaA, Darmstadt, Germany), which distinguishes between the following four populations of cells: non-apoptotic, early apoptotic, late stage apoptotic and dead cells. Namely, as described in our previous study cited here [23], the assay was performed according to the manufacturer’s protocol, where A549 cells were seeded in 12-well plates two days prior to the experiment at a density of 30,000 cells/well. On the day of the experiment, growth medium was replaced with 500 µL of fresh unsupplemented medium containing PBDEs at 12 µg mL^−1^ concentration for a 4 h incubation at 37 °C in a humidified atmosphere containing 5% CO_2_. Thereafter, media-containing tested compounds were removed, and adherent cells were washed with PBS and detached using 0.25% Trypsin/EDTA solution (Sigma-Aldrich, Steinheim, Germany). Trypsin was inactivated with 500 µL of complete media (supplemented with 5% FBS), and cell suspension was transferred to Eppendorf tubes. A volume of 80 µL of cell suspension was mixed with 100 µL of Annexin V reagent (The Muse™ Annexin V & Dead Cell Assay, Merck KGaA) in a new tube and incubated in dark for 30 min at room temperature. Each experiment was performed at least two or three times, in duplicate. Measurement, as well as analysis, was performed on the flow cytometer Muse™ Cell Analyzer (Luminex, Poland). Paraformaldehyde (PFA, Sigma-Aldrich, Steinheim, Germany), 0.08% final concentration, was used as a positive control. Data were evaluated by Muse™ and Prism software and presented as a percentage of control.

### 2.9. In Silico Predictions of CYP Inhibition

CYP inhibition was estimated in silico by validated online platform SwissADME [26]. The model is built on a training set of: 9145 and tested on 3000 molecules for CYP1A2; 9271 and tested on 3000 molecules for CYP2C19; 5940 and tested on 2075 molecules for CYP2C9; 3664 and tested on 1068 molecules for CYP2D6; and 7518 and tested on 2579 molecules for CYP3A4.

### 2.10. Statistics

Results are presented as means of three independent experiments in duplicate with a standard error (SD), unless stated otherwise. In the results, n refers to the number of replicas used in the calculation for the statistics. One-way ANOVA followed by Dunnett’s test were performed to test for differences between the groups and untreated control. For evaluation of intracellular signaling, unpaired t-test was used. Statistical analyses were performed using the Prism software. Statistical significance (*p*-value) spanned from <0.05 to <0.0001.

## 3. Results

### 3.1. Cell Viability

**Figure 2:**
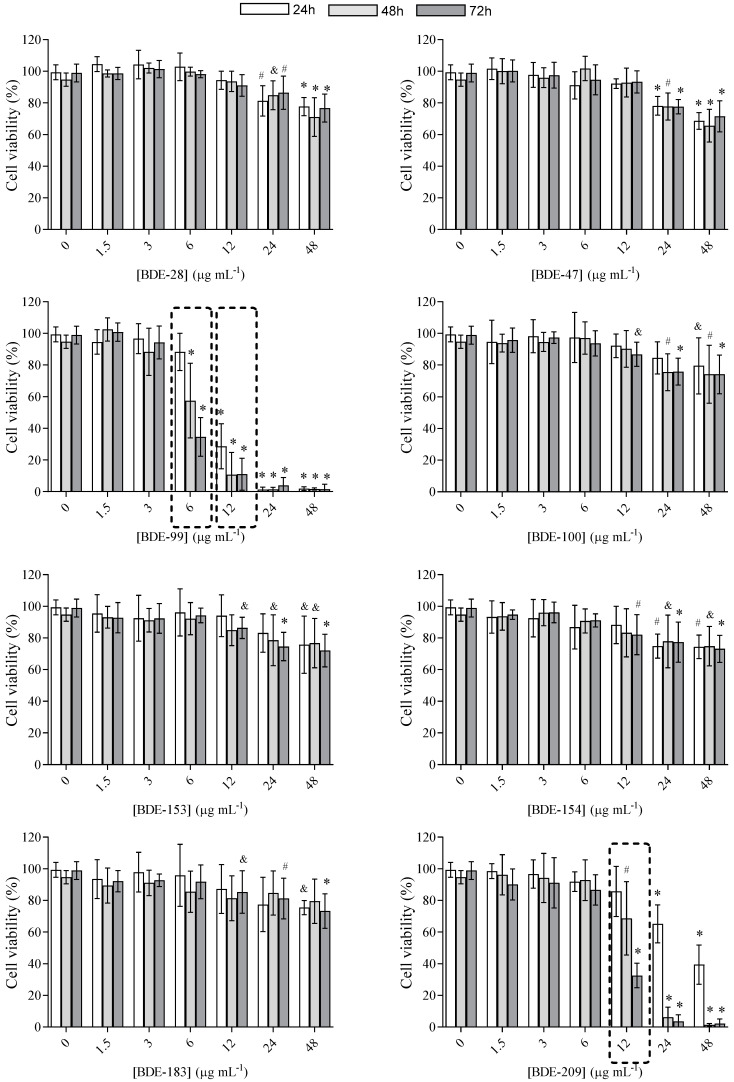
Viability of A549 cells after prolonged (24, 48 and 72 h) exposure to PBDEs at concentrations from 1.5 to 48 μg mL^−1^ determined with the MTS assay; 0 μg mL^−1^ represents untreated cells. The results are expressed as percentage of corresponding control, untreated cells and given as means ± SD (*n* = 6). The dashed frame highlights a time-dependent effect. & *p* < 0.05; # *p* < 0.01; * *p* < 0.0001 vs. untreated control.

A549 cells were exposed to each PBDE congener tested at concentrations of up to 48 µg mL^−1^ for 24, 48 and 72 h, as we wanted to simulate prolonged exposure to accumulated PBDEs in real samples. The obtained results are summarized in Figure 2. All of the congeners affected A549 cell viability, and a statistically significant effect was observed at higher congener concentrations. A significant time-dependent effect was observed only for congeners BDE-99 and BDE-209, starting from concentrations of 6 or 12 µg mL^−1^ and killing nearly all cells at 48 µg mL^−1^ in 72 h. For the rest of the tested PBDEs, cell survival dropped 15–30% compared to the control within 24–72 h.

**Figure 3:**
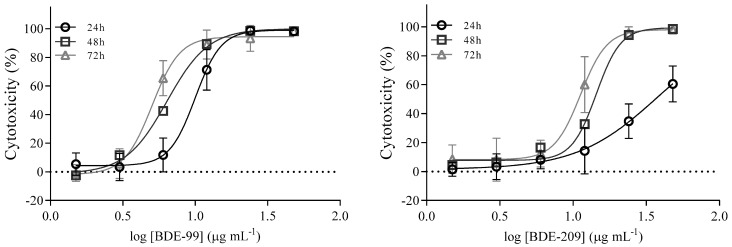
Cytotoxicity curves of BDE-99 and -209 at concentrations from 1.5 to 48 μg mL^−1^ in A549 cells over 24, 48 and 72 h. The results of the MTS assay are given as means ± SD (*n* = 6).

Furthermore, for BDE-99 and BDE-209, IC_50_ values were determined, and results are shown in Figure 3 and Table 1. As can be seen, cytotoxicity increased up to 3.5-fold over time, meaning that even lower concentrations killed cells at a longer exposure time.

**Figure 4:**
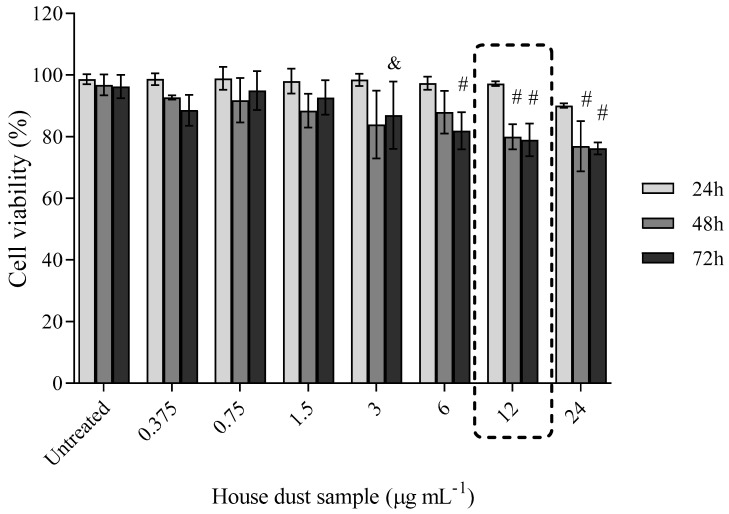
Prolonged exposure of A549 cells to a house dust sample extract. A549 cells were exposed to ∑PBDE in house dust at concentrations from 0.375 to 24 μg mL^−1^ for 24, 48 and 72 h, and cell viability was determined with the MTS assay. The results are given as means ± SD (*n* = 6). The dashed frame highlights the concentration found in the house dust sample. & *p* < 0.05; # *p* < 0.01; vs. untreated control.

Results obtained for cell exposure to a PBDE mixture from a real-life house dust sample are presented in Figure 4. A significant drop in A549 cell viability was observed with exposure to 12 and 24 μg mL^−1^ for 48 and 72 h.

#### Cell Viability after Exposure to BDE-99 and -209 Mixture

**Figure 5:**
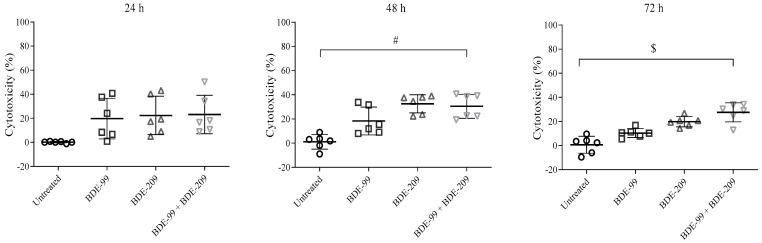
LOAEL cytotoxicity of BDE-99 (7.5 μg mL^−1^) and BDE-209 (15 μg mL^−1^), alone and combined, in A549 cells treated for 24, 48 and 72 h. The results of the MTS assay are given as means ± SE (*n* = 6). # *p* < 0.01; $ *p* < 0.001 vs. untreated control.

To determine whether an effect of reducing cell viability could be a result of the additive action of PBDEs present in the house dust sample, the cells were exposed to a mixture of two of the most toxic PBDE congeners in their respective LOAEL (Low Observed Adverse Effect Level) concentrations, i.e., the concentrations reaching IC_20_ at 24 h (BDE-99 of 7.5 μg mL^−1^ and BDE-209 of 15 μg mL^−1^). Results are given in Figure 5. With the prolonged time of exposure, no significant decrease in cell viability compared to individual exposures was observed, meaning that neither a synergistic, nor additive, nor antagonistic effect was detected.

### 3.2. Cell Status in the Presence of PBDEs

We determined changes in A549 membrane integrity, reactive oxygen species (ROS), intracellular glutathione (GSH) and mitochondrial membrane potential (ΔΨm) status 4 h after exposure to three concentrations of each PBDE congener: 12 μg mL^−1^—the highest concentration of a single BDE congener (BDE-99) found in the real house dust sample extract; 6 μg mL^−1^—concentration that affected cell viability in viability assay; and 3 μg mL^−1^—concentration that did not affect cell viability significantly for most of the congeners tested. In addition, we determined whether these compounds initiated regulated cell death, i.e., apoptosis. For these experiments, only the highest concentration was used, as it was the only one causing significant damage to the cells.

#### 3.2.1. BDE-99, -153, -154 and -209 Affect Cell Membrane Integrity

**Figure 6:**
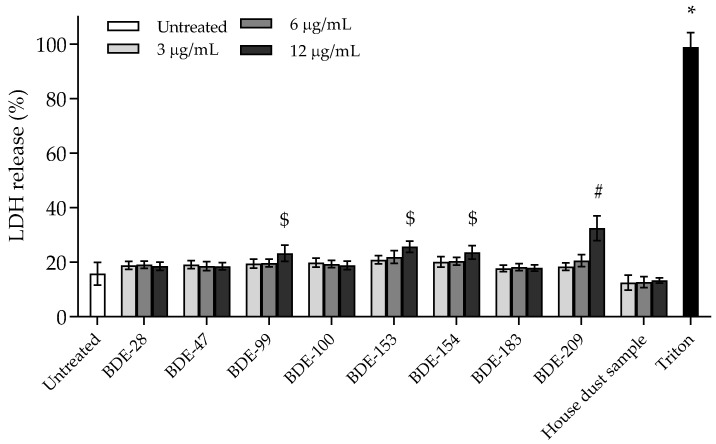
Levels of LDH release after 4 h exposure of A549 cells to PBDE congeners and house dust sample extract. Triton (0.08%) was used as the positive control. The results are presented as percentage of LDH release and given as means ± SD (*n* = 3). # *p* < 0.01; $ *p* < 0.001; * *p* < 0.0001 vs. untreated control.

Cell membrane integrity, as an indicator of cell rupture or necrosis, was determined by measuring the release of the intracellular enzyme lactate dehydrogenase (LDH) from cells with a damaged membrane into the medium after exposure to PBDEs. However, LDH as a marker has a limited stability in cell medium and will be degraded by specific proteases present (*CytoTox-ONE™ Homogeneous Membrane Integrity Assay*-Instructions for Use of Products). In this study, we therefore decided to investigate the 4 h exposure time point to ensure adequate LDH detection [27]. Additionally, at that time point, cells that entered the late apoptosis stage (secondary necrosis) could be detected. Results are presented in Figure 6. Within the exposure time of 4 h, a statistically significant percentage of LDH in the cell medium was detected only for BDE-99, -153, -154 and -209, applied at the highest concentration. The obtained increase of 20% compared to control is in accordance with the obtained results for cytotoxicity and indicates their probable mechanism of action. The other PBDEs did not increase the presence of LDH during the 4 h exposure period.

#### 3.2.2. PBDEs Induce ROS

**Figure 7:**
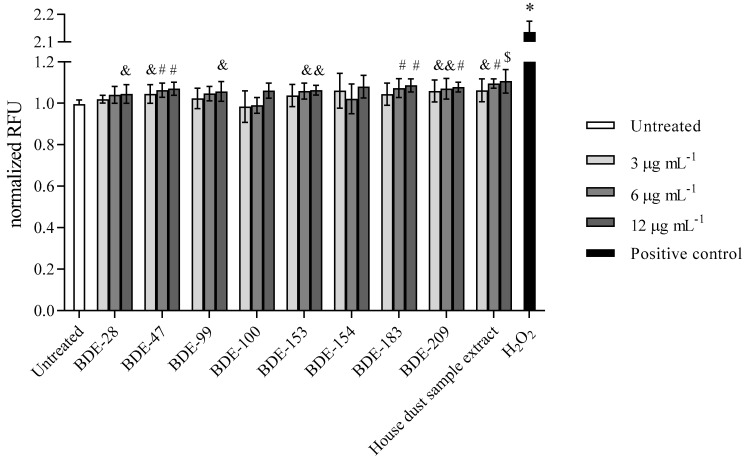
ROS levels after the 4 h exposure of A549 cells to PBDE congeners, house dust sample extract and H_2_O_2_ (100 µM) as positive control. The results of DCF-fluorescence signal measurements are presented as relative fluorescence units (RFU) and given as means ± SD (*n* = 6). & *p* < 0.05; # *p* < 0.01; $ *p* < 0.001; * *p* < 0.0001 vs. untreated control.

Results obtained for ROS induction in the presence of PBDEs are presented in Figure 7. According to the statistical method applied, a significant rise in ROS level within 4 h of exposure was observed in treatment with congeners BDE-28 and -99 at 12 μg mL^−1^, with BDE-153 and -183 at 6 and 12 µg mL^−1^, and with BDE-47 and -209 at all of the concentrations applied. Interestingly, BDE-100 and -154 did not increase the ROS (Figure 6). As expected from the above findings, exposure to the house dust extract increased ROS production significantly.

#### 3.2.3. PBDEs Decrease GSH Levels

**Figure 8:**
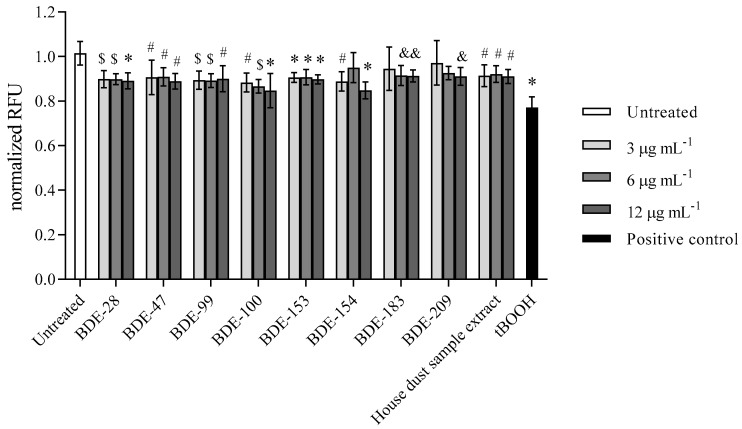
GSH levels after the 4 h exposure of A549 cells to single PBDEs and house dust sample extract at 3, 6 and 12 µg mL^−1^ and to tBOOH (100 µM) as positive control. The results of MCB-fluorescence measurements are presented as relative fluorescence units (RFU) and given as means ± SD (*n* = 6). & *p* < 0.05; # *p* < 0.01; $ *p* < 0.001; * *p* < 0.0001 vs. untreated control.

Results obtained for glutathione (GSH) levels as an antioxidant with a protective role against high levels of ROS are presented in Figure 8. A statistically significant drop in GSH level was detected for all of the tested PBDE congeners within 4 h of exposure. The only exception observed in lower concentrations of BDE-183 and -209 could be explained by the higher statistical deviation of the measured signal.

#### 3.2.4. BDE-100, -154 and -209 Reduce Mitochondrial Membrane Potential

**Figure 9:**
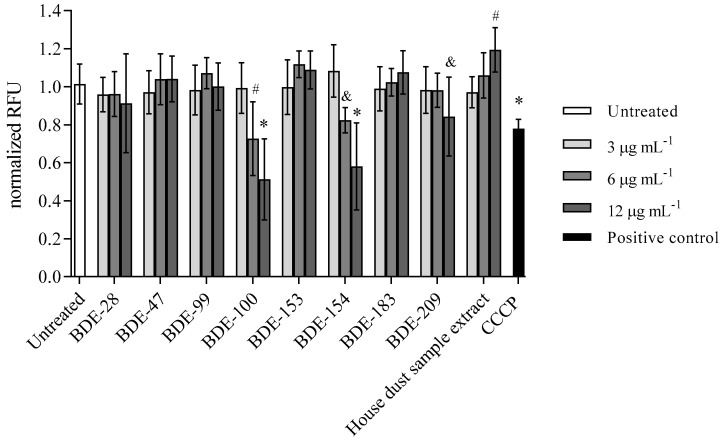
Mitochondrial membrane potential after 4 h exposure of A549 cells to single BDE congeners and house dust sample extract at 3, 6 and 12 µg mL^−1^ and CCCP (50 µM) as positive control. The results are presented as relative fluorescence units (RFU) and given as means ± SD (*n* = 6). & *p* < 0.5, # *p* < 0.01, * *p* < 0.001 vs. untreated control.

The results on the disruption of the mitochondrial membrane potential as another trigger of the cell death mechanism are shown in Figure 9. The most significant loss of mitochondrial membrane potential was observed for treatment with BDE-100, -154 and -209, but, interestingly, the treatment with house dust sample extract increased it at a statistically significant level (Figure 9).

#### 3.2.5. BDE-28 Activates Apoptosis

**Figure 10:**
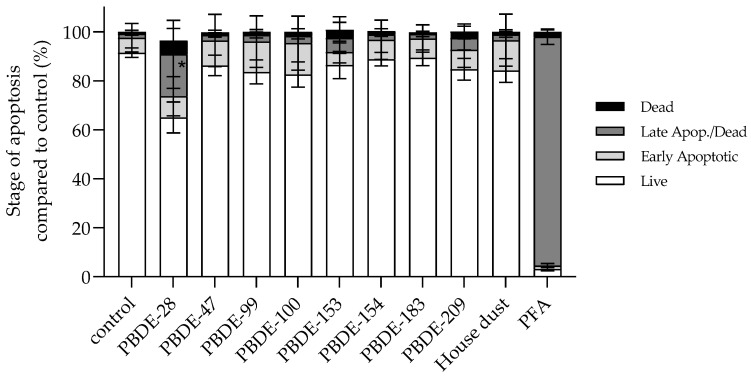
Percentage of different stages of apoptosis of A549 cells after 4 h exposure to single PBDE congener and house dust sample extract at 12 µg mL^−1^ and PFA (0.08%, *v*/*v*) as a positive control. The results are presented as percentage of total apoptotic cells compared to vehicle control (2.4% methanol) and given as means ± SD (*n* = 6). * *p* < 0.001 vs. control.

As all the observed PBDE-induced disturbances determined A549 homeostasis, the initiation of regulated cell death, i.e., apoptosis, was also checked. According to the results shown in Figure 10, after 4 h of exposure, a significant increase in late apoptotic events was noticed only after exposure to BDE-28. However, a tendency of potential increase in early apoptotic events was noted for -47, -99, -100 andfor house dust sample, and in total apoptotic events for -47, -153 and -209.

### 3.3. Predicted CYP Enzyme Inhibition by PBDEs

According to the in silico prediction (Table 2), all of the congeners, except for BDE-28, were not expected to inhibit the majority of metabolic CYP enzymes, irrespective of the number of bromine atoms present. The aim was to establish whether there is a possibility that PBDEs affect the metabolism once they enter the body, thus influencing their metabolism and prolonging circulation time or inhibiting the system itself, which can lead to toxicity and cell death. The exception was determined only for CYP2C9, predicted to interact with all of the studied congeners. Interestingly, BDE-28, as the simplest of the tested congeners, showed the possibility of a broader CYP enzyme inhibition. Nevertheless, such potential inhibition of certain CYPs needs to be investigated and confirmed in further in vitro studies.

## 4. Discussion

The main goal of this study was to examine the effects of eight BDE congeners found in real house dust, including the well-known BDE-47, -99 and -209, as well as the less studied ones, such as BDE-28, -100, -153, -154 and -183. In our experimental approach, we simulated a prolonged exposure to cells (up to 72 h) to mimic, in a way, the real-life conditions of exposure to these compounds.

As determined by our study, the toxicity of these compounds on A549 lung cancer cells varied depending primarily on the concentration applied, but likewise, the structure of the congeners influenced it in some part as well. In that aspect, the position of bromine atoms played a greater role than their overall number in the compounds’ structure. This was seen, for example, from the observed differences in the toxicity of specific analogs BDE-99 and BDE-100 with five bromine atoms. Interestingly, only for the two most toxic congeners, BDE-99 (5 Br atoms) and BDE-209 (10 Br atoms), was time-dependent toxicity observed, meaning that even smaller concentrations of these compounds will have negative effects over time. This time-dependent toxicity was confirmed with a tested house dust sample that contained these congeners. Additionally, as our results indicated, it appears that the action of the congeners in the mixture will not be additive or synergistic, but the effects of the one present in the highest concentration will prevail. This could be indicative with regard to the constant exposure to a mixture of PBDE congeners through different exposure pathways and thereby presenting a risk for human health.

All of the tested congeners have been shown to affect cell homeostasis at the higher concentrations applied. For a detailed analysis, cells were exposed to PBDEs for 4 h to capture the initial activation of various targets involved in toxicity, which would lead to cell death within 24–72 h. Results indicate that exposure to most PBDEs leads to oxidative stress and cell damage, probably by disruption of the oxidative phosphorylation chain, and with some PBDEs, to the disruption of the mitochondrial membrane potential, which provides the necessary energy in cells [13]. This is also in accordance with several previous studies [6,8,9,18]. Higher levels of ROS cause damage to proteins, nucleic acids, lipids, membranes and organelles, which can lead to the activation of cell death processes, such as apoptosis [28,29]. However, in this study the induction of apoptosis was detected only after treatment with BDE-28. Since we observed induction of ROS with this congener, this suggests the activation of apoptosis, but not by mitochondrial induction pathway, since no effect was observed on mitochondria. The release of LDH was observed for BDE-99, -153, -154 and -209, which indicates that cell breakage or necrosis occurred, rather than apoptosis, within the time frame tested. Nevertheless, other studies showed the activation of apoptotic markers by BDE-209 and the release of LDH, suggesting death by secondary necrosis [30]. Chen et al. also showed induced apoptosis by BDE-209 via the mitochondrial signaling on the neuronal Neuro-2a cell line. Other studies showed that BDE-153 also activates apoptosis, which is manifested as late apoptosis or secondary necrosis, with activation of apoptosis-inducing factor (AIF) and caspase 3 [10], directly affecting cell survival [31,32].

In our study, as opposed to other congeners, BDE-100 did not show necrotic, only early apoptotic effects within 4 h, but it lowered mitochondrial membrane potential.

Overall, the observed specific effects of PBDEs could also be triggered by interactions with receptors on the cell surface. It has been shown that PBDEs interact, for example, with thyroid receptors [33], as well as gamma-aminobutyric acid (GABA) [34], vascular endothelial growth factor (VEGF) [35] or different hormone receptors [36]. Binding to receptors can trigger cascade reactions that can lead to disruptions of the mitochondrial membrane potential and therefore induce production of ROS [36].

In general, our results confirmed previous findings, and in addition, showed that prolonged in vitro exposure to tested PBDEs affects lung cells, which suggests that it could also affect human lungs in real organism. Therefore, it is important to monitor the impact of prolonged exposure, as signs of chronic toxicity may occur later on [37]. However, we are aware that it is difficult to simply translate one’s in vitro findings to in vivo situations. In the living organism, PBDEs bind to hydrophobic molecules normally present in blood, human milk or tissue (especially adipose tissue). As such, they bind with lipids in the body and can be distributed (and metabolized in the liver), or even released from the tissue, for example upon weight loss or lactation [4,38,39]. However, according to the in silico prediction, the tested PBDEs could inhibit important CYP2C9 enzyme and thus influence their metabolism and prolong circulation time, but this needs to be verified in future studies.

The presence of PBDEs in the environment and impact on human health will surely be studied in the years to come. The high overall PBDEs concentrations present in house dust samples, like the one found recently in Zagreb, Croatia, and the one tested here, demand search for potential pollution sources and warrant a monitoring of the long-term influence of PBDEs on human health. Additionally, this could be of particular interest in countries such as the UK, the US and Canada, where concentrations detected in dust samples are still generally high, due to very strict fire protection regulations [40,41].

## 5. Conclusions

In this study, in vitro cell-based assays on human carcinoma alveolar basal epithelial cells A549 cells indicated the activation of unwanted effects of less studied PBDE congeners, namely BDE-28, -100, -153, -154 and -183. A decrease in cell viability was caused by PBDEs stimulated induction of ROS, GSH consumption, loss of cell membrane integrity, disturbance in mitochondrial membrane potential and eventually either regulated or unregulated cell death. For the two most toxic congeners, the toxic effects were time dependent, meaning that even smaller concentrations of these compounds will have negative effects over time. Therefore, in the cases of high ΣPBDE concentrations in house dust, where a mixture of such toxic congeners is present, continuous and prolonged exposure to PBDEs might lead to adverse effects on human health. Furthermore, the low toxicity observed for other tested congeners in the studied concentration and time frame does not exonerate these chemicals, and further studies to evaluate them are warranted, such as, for example, the effects after longer exposure periods.

## Figures and Tables

**Table 1:** IC_50_ values of BDE-99 and -209 in A549 cells over 24, 48 and 72 h.

Time (h)	IC_50_ ± SD (µg mL^−1^) BDE-99	IC_50_ ± SD (µg mL^−1^) BDE-209
24 h	9.8 ± 1.1	37.2 ± 2.9
48 h	6.6 ± 1.1	13.8 ± 1.1
72 h	5.3 ± 1.1	11.0 ± 1.1

**Table 2:** In silico cytochrome P450 (CYP) inhibition.

CYP	BDE-28	BDE-47	BDE-99	BDE-100	BDE-153	BDE-154	BDE-183	BDE-209
1A2	+	-	-	-	-	-	-	-
2C19	+	-	-	-	-	-	-	-
2C9	+	+	+	+	+	+	+	+
2D6	-	-	-	-	-	-	-	-
3A4	-	-	-	-	-	-	-	-

“-“ predicted inability to inhibit CYP; “+” predicted to inhibit CYP.

## Data Availability

Not applicable.
